# Retraction: Longitude variation of the microRNA-497/FGF-23 axis during treatment and its linkage with neoadjuvant/adjuvant trastuzumab-induced cardiotoxicity in HER2-positive breast cancer patients

**DOI:** 10.3389/fsurg.2023.1307330

**Published:** 2023-10-11

**Authors:** Frontiers Editorial Office

A Retraction of the Original Research Article Longitude variation of the microRNA-497/FGF-23 axis during treatment and its linkage with neoadjuvant/adjuvant trastuzumab-induced cardiotoxicity in HER2-positive breast cancer patients By Liu H, Hu X, Wang L, Du T, Feng J, Li M, Liu L and Liu X (2022) Front. Surg. 9:862617. doi: 10.3389/fsurg.2022.862617

The journal and Chief Editors retract the 11 May 2022 article cited above.

This article is retracted by Frontiers. The publisher has discovered that the authors provided false information for the peer-review process. As the scientific integrity of the article cannot be guaranteed, and adhering to the recommendations of the Committee on Publication Ethics (COPE), the publisher therefore retracts the article.

The authors have not responded to this retraction.

This retraction was approved by the Chief Editors of Frontiers in Surgery and the Chief Executive Editor of Frontiers.

